# Influence of Climate on Emergency Department Visits for Syncope: Role of Air Temperature Variability

**DOI:** 10.1371/journal.pone.0022719

**Published:** 2011-07-27

**Authors:** Andrea Galli, Franca Barbic, Marta Borella, Giorgio Costantino, Francesca Perego, Franca Dipaola, Francesco Casella, Pier Giorgio Duca, Andrè Diedrich, Satish Raj, David Robertson, Alberto Porta, Raffaello Furlan, on behalf of the STePS Investigators

**Affiliations:** 1 Emergency Department, Vimercate Hospital, Vimercate, Milan, Italy; 2 Neuroscience Research Association, Internal Medicine, “Bolognini” Hospital, Seriate, Bergamo, Italy; 3 Internal Medicine 2, “L. Sacco” Hospital, Milan, Italy; 4 Internal Medicine 3, “L. Sacco” Hospital, Milan, Italy; 5 Internal Medicine, Sesto S. Giovanni Hospital, Sesto S. Giovanni, Milan, Italy; 6 Medical Statistics, Institute of Clinical Science “L.Sacco”, Milan, Italy; 7 Division of Clinical Pharmacology, Department of Medicine, Autonomic Dysfunction Center, Vanderbilt University, Nashville, Tennessee, United States of America; 8 Department of Technologies for Health, Galeazzi Orthopaedic Institute, Milan, Italy; 9 University of Milan, Milan, Italy; Heart Center Munich, Germany

## Abstract

**Background:**

Syncope is a clinical event characterized by a transient loss of consciousness, estimated to affect 6.2/1000 person-years, resulting in remarkable health care and social costs. Human pathophysiology suggests that heat may promote syncope during standing. We tested the hypothesis that the increase of air temperatures from January to July would be accompanied by an increased rate of syncope resulting in a higher frequency of Emergency Department (ED) visits. We also evaluated the role of maximal temperature variability in affecting ED visits for syncope.

**Methodology/Principal Findings:**

We included 770 of 2775 consecutive subjects who were seen for syncope at four EDs between January and July 2004. This period was subdivided into three epochs of similar length: 23 January–31 March, 1 April–31 May and 1 June–31 July. Spectral techniques were used to analyze oscillatory components of day by day maximal temperature and syncope variability and assess their linear relationship.

There was no correlation between daily maximum temperatures and number of syncope. ED visits for syncope were lower in June and July when maximal temperature variability declined although the maximal temperatures themselves were higher. Frequency analysis of day by day maximal temperature variability showed a major non-random fluctuation characterized by a ∼23-day period and two minor oscillations with ∼3- and ∼7-day periods. This latter oscillation was correlated with a similar ∼7-day fluctuation in ED visits for syncope.

**Conclusions/Significance:**

We conclude that ED visits for syncope were not predicted by daily maximal temperature but were associated with increased temperature variability. A ∼7-day rhythm characterized both maximal temperatures and ED visits for syncope variability suggesting that climate changes may have a significant effect on the mode of syncope occurrence.

## Introduction

Syncope is a clinical event characterized by a transient loss of consciousness, estimated to affect 6.2/1000 person-years [1], resulting in remarkable health care costs and indirect social costs due to the loss of working hours. Conventional physiology teaching suggests that heat may facilitate orthostatic intolerance and syncope during standing. The available evidence [2]–[4] can be summarized as follows. Whole-body heat stress results in cutaneous vasodilatation aimed at heat dissipation ultimately leading to a reduction in cardiac ventricular filling pressure [5], decrease in central blood volume [5] and central venous pressure [4]. A concomitant orthostatic challenge leads to blood pooling in the limb and splanchnic vascular bed [6], and likely to a further decline of central venous pressure [7]. This might critically impact cardiac filling thus overwhelming the peripheral vasoconstriction required to maintain arterial blood pressure and cerebral blood flow on standing [6]. These responses may eventually lead to syncope.

However, studies designed for public health surveillance, analyzing the changes in heat-related morbidity associated with seasonal high temperatures or heat weaves, only indirectly [8]–[11] and inconsistently [8] corroborated the hypothesis that high environmental temperatures may promote syncope. Notably, most of those studies did not evaluate the effects of the normal seasonal increase of temperature but focused on the hottest months and the consequences on health of the heat waves.

Furthermore, there are remarkable day by day fluctuations (i.e. variability) in maximal temperature within an overall progressive increase from winter to summer but the potential role of temperature variability in affecting human adaptation to heat and its relationship with syncope onset has never been evaluated.

Using the database of the STePS study [12] we tested the hypothesis that the increase of temperatures from January to July might be associated with a progressive increase in the number of ED visits for syncope. As a second aim we focused on both daily maximal temperature and syncope variability in order to assess their potential relationship.

## Methods

### Population

Inclusion criteria: the present study used the STePS study [12] database. This multi-center investigation evaluated the short and long term prognoses of patients who were seen for syncope at the ED of four Italian hospitals in the Milan area between January 23 and July 31, 2004. Consecutive subjects older than 18 years of age were included.

The following exclusion criteria must not have been present: 1) a referred head injury preceding the loss of consciousness; 2) non spontaneous return to consciousness; 3) non-syncopal syndromes such as vertigo, coma, shock, seizure; 4) recent alcohol or drug abuse; 5) unwillingness to provide informed consent.

Among 2775 screened individuals, inclusion criteria [12] were satisfied by 770 patients who were enrolled in the study.

The study was approved by the Ethical Committee on Human Research of the Coordinating Centre (Hospital “L. Sacco”), and all participants provided written or verbal [12] informed consent. Verbal consent was obtained during a phone interview in those patients that were discharged from ED, according to the prospective observational design of the study and the Ethical Committee approval.

### Definitions

Syncope was defined as a transient loss of consciousness due to cerebral hypoperfusion characterized by rapid onset, short duration and spontaneous recovery [13].

### Study end points

The first aim of the present study was to assess the relationship between the increase of temperatures from January to July and the potential increase in the rate of ED admission for syncope. In order to evaluate whether hot months were associated with different rate of syncope compared to cold months, the observation period was subdivided into three consecutive epochs of similar length. Epoch #1 lasted from January 23^rd^ to March 31^st^ (69 days), epoch #2 from April 1^st^ to May 31^st^ (61 days) and epoch #3 from June 1^st^ to July 31^st^ (61 days). The number of ED visits for syncope corresponding to each epoch was assessed both in absolute numbers and after normalization by corresponding total ED accesses, and further evaluated in sub-groups of patients characterized by different age and gender.

Our second aim was to address maximal temperature and syncope ED visits variability and assess their potential relationship. The working hypothesis was that if ambient temperatures affected syncope onset then the maximal temperatures spontaneous fluctuations should be mirrored by analogous and correlated oscillations in the pattern of ED visits for syncope.

### Weather indices

**Figure 1:**
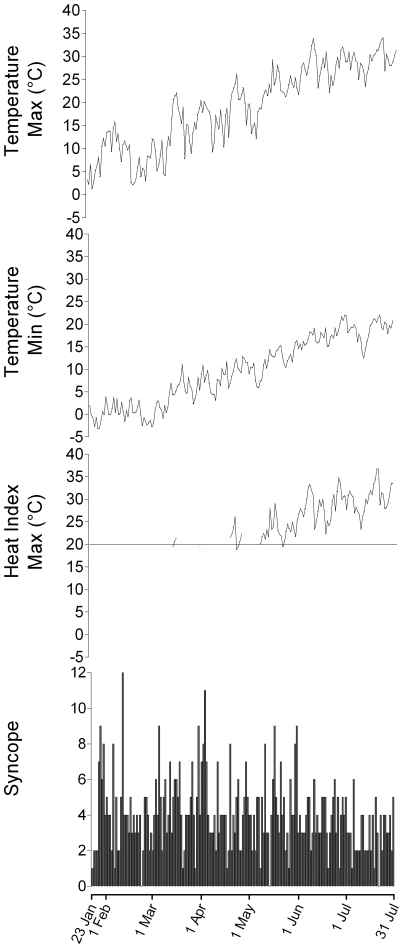
Day by day values of maximal and minimal air temperature, heat index and of syncope observed from January 23^rd^, 2004 to July 31^st^, 2004. The expected progressive increase of air temperature from January diverged from Emergency Department (ED) visits for syncope which remained stable until May, before decreasing. Maximal and minimal air temperatures fluctuate (temperature variability) on a day by day basis. The temperature variability was lower in June and July compared to the cooler months. Heat index has been computed only for values of maximal temperature >20°C and its spontaneous variability mirrors maximal temperature fluctuations.

Weather indices were provided by the Centro Metereologico Lombardo. A digital thermo-humidity sensor SHT11 (Sensirion Instr. Zurich, Switzerland) was used to assess temperature and humidity. Maximal and minimal air temperatures, dew point and relative humidity were sampled every 10 minutes every day from January 23^rd^ 2004 to July 31^st^ 2004 by means of a Davis Vantage Pro 2 weather station (Davis Instr., Hayward, Ca) located in Sedriano (Milan, Italy). The Heat Index, which evaluates the perceived temperature, was computed from daily maximal temperature and relative humidity according to the Steadman approach [14]. Heat Index values were calculated only for air maximal temperature ≥20°C (Figure 1).

### Temperature and syncope variability

Daily maximal and minimal temperature values and the number of syncope were sampled once per day, thus obtaining the corresponding series. The series were analyzed in the frequency domain by means of an autoregressive spectral method [15]. The normalized frequency of the power spectrum ranged from 0 to 0.5 cycles×day^−1^. The power spectral density was expressed as °C^2^ × day^−1^× cycles^−1^ in the case of the series of the maximal and minimal temperatures and as N^2^× day^−1^× cycles^−1^ in the case of the series of the syncope rate. The frequency of the dominant oscillation (DO) detected in the power spectrum was extracted (i.e. f_DO_) and its period was calculated as f_DO_^−1^ (measured in days). All series were linearly de-trended before applying spectral analysis in order to filter out the seasonal trends.

The deterministic nature of the oscillations was tested using a surrogate data approach [16]. The original series were shuffled, thus preserving the distribution of the series but fully destroying their autocorrelation. A set of 100 surrogates was created from each original series and the power spectral density (PSD) was estimated over each surrogate. At any given frequency the PSD distribution and the 95^th^ percentile were calculated. The 95^th^ percentile, as a function of the normalized frequency, was taken as the threshold for assessing the deterministic nature of the oscillation [17]. If the PSD calculated over the original series was above this threshold at a given frequency (i.e. above the level defined by a white noise with the same distribution of the original series), that specific oscillation was deemed to be significantly present.

We utilized the squared coherence function to measure the degree of linear correlation between two series as a function of the frequency. It ranged from 0 (no correlation) to 1 (perfect correlation). Squared coherence was estimated using a bivariate autoregressive approach [15] with a model order fixed to 10. The deterministic nature of the linear link between two oscillations present in two series at the same frequency was tested using again a surrogate data approach [18] (see before). The squared coherence function was calculated over each pair of surrogates. At any given frequency we constructed the distribution of the coherence values and the 95^th^ percentile was calculated. The two series were significantly coupled at that specific frequency [19] if the coherence was above the threshold (i.e. course of the 95^th^ percentile) at a given frequency.

In order to compare the air temperature variability values corresponding to each of the three epochs, the mean ± SD of the differences between daily maximum and minimum values of air temperature was calculated during the corresponding epoch.

### Statistical analysis

A linear correlation was used to assess the relationship between daily maximal temperature values and number of syncope. All analyses were two-tailed, and p values<0.05 were considered significant.

Descriptive statistics were used for categorical variables. Differences were evaluated by the chi-square test.

Maximal temperature values were expressed as mean±SD. Differences in the maximal temperature and temperature variability corresponding to the three epochs were evaluated by one-way ANOVA and Bonferroni or Dunnett corrections for post-hoc tests.

## Results

The demographic features and the clinical characteristics of the study population are summarized in Table 1.

**Table 1:** Demographic and clinical features of the population studied.

Age ± SD, y	51±22
18–44 y	250 (32.0)
45–65 y	308 (40.0)
>75 y	212 (28.0)
Gender	
Women	431 (56.0)
Men	339 (44.0)
Past medical history	
Hypertension	275 (39.9)
Structural heart disease	174 (25.3)
Heart failure	30 (4.4)
Ventricular arrhythmias	12 (1.7)
Cerebrovascular diseases	91 (13.2)
Neurological diseases	65 (9.4)
Diabetes mellitus	69 (10.0)
COPD	54 (7.8)
Neoplasias	55 (8.0)
Index syncope circumstances	
Supine/Sitting	160 (23.2)
Upright posture	514 (74.6)
During exercise	15 (2.2)
First episode	296 (43.0)
Trauma	166 (24.1)
Abnormal ECG at presentation	232 (33.7)
Absence of preceding symptoms	195 (28.3)

Values expressed as mean±SD or n (%). Past medical history, index syncope circumstances, trauma, abnormal ECG at presentation and absence of preceding symptoms refer to 689 subjects because 81 patients had incomplete medical and index syncope history. COPD indicates Chronic Obstructive Pulmonary Disease.

### ED visits for syncope and temperature increase from January to July

Figure 1 depicts the daily admissions to ED for syncope and the corresponding maximal and minimal air temperatures (°C) and Heat Index (for maximal temperatures >20°C) observed on a daily basis from January 23^rd^ to July31^st^. Maximal and minimal temperature changes showed a high variability characterized by volleys of mild increases over days followed by slow and incomplete declines over days within the expected progressive enhancement from January to July. The Heat Index mirrored the maximal air temperature pattern.

Daily ED visits for syncope were fairly constant from January to May. Thereafter, number of syncope declined (Figure 1), although both the maximal temperature and the heat index reached the highest values. There was no correlation (r^2^ = 0.0089, p = N.S.) between the daily maximal temperatures and the number of ED visits for syncope.

### Reduced frequency of ED visits for syncope during June and July

Table 2 shows syncope number and percentage during the three consecutive epochs resulting from the subdivision of the period of observation.

**Table 2:** Modifications of the rate of syncope, in all the patients who presented to ED for syncope and in subpopulations of different age and gender, grouped according to three epochs.

	EPOCH 1	EPOCH 2	EPOCH 3
	Jan–Feb–Mar(69 days)	Apr–May(61 days)	Jun–Jul(61 days)
Max Temperature, °C	10.0±5.1(range 1.2–22.1)	20.0±4.4#(range 9.2–29.2)	28.6±2.8§(range 21.7–34.0)
Max Temperature Variability, °C	8.8±5.1	10.8±4.4	6.5±2.8§
Total ED visits, n	34608	31289	31938
Syncope ED visits, n (‰)	296 (8.6)	267 (8.5)	207 (6.5)*
Admitted Syncope, n (%)	104 (35.1)	98 (36.7)	71 (34.3)
Syncope aged >75 y, n (%)	82 (27.7)	75 (28.1)	55 (26.6)
MaleTotal Syncope, n (%)	133 (44.9)	111 (41.6)	95 (45.9)
Syncope aged >75 y, n (%)	34 (25.6)	23 (20.7)	23 (24.2)
FemaleTotal Syncope, n (%)	163 (55.1)	156 (58.4)	112 (54.1)
Syncope aged >75 y, n (%)	48 (29.4)	52 (33.3)	32 (28.6)

Max Temperature is the mean ± SD of daily Maximal Temperatures. Max Temperature Variability is the mean ± SD of day by day Maximal Temperatures variations in each epoch. Admitted Syncope refers to patients admitted to hospital for syncope. n is the number; ‰ is the number of Syncope ED visits per thousand, in respect to Total ED visits; % is the percentage of Admitted Syncope in respect to Syncope ED visits and of Syncope aged >75 yrs in respect to Syncope ED visits.

*p<0.05 vs epoch 2 and epoch 1;

§ p<0.001 vs epoch 2 and epoch 1;

# p<0.001 vs epoch 1.

Mean maximal air temperature was significantly (p<0.001) higher in epoch 3 (28.6±2.8°C, p<0.01) compared to epoch 1 (10.0±5.1°C) and epoch 2 (20.0±4.4°C), and in epoch 2 compared to epoch 1 (p<0.01). Conversely, maximal temperature variability was significantly lower in epoch 3 compared to the other two epochs (Table 2).

ED visits for syncope, when normalized by total ED visits, was lower (p<0.05) in epoch 3 compared to epoch 1 and epoch 2 (Table 2).

Hospital admission for syncope was similar in the three epochs (Table 2). When analyzed by gender and age, ED visits for syncope were unaffected by the different maximal temperatures (Table 2).

### Relationship between temperature and ED visits for syncope variability

**Figure 2:**
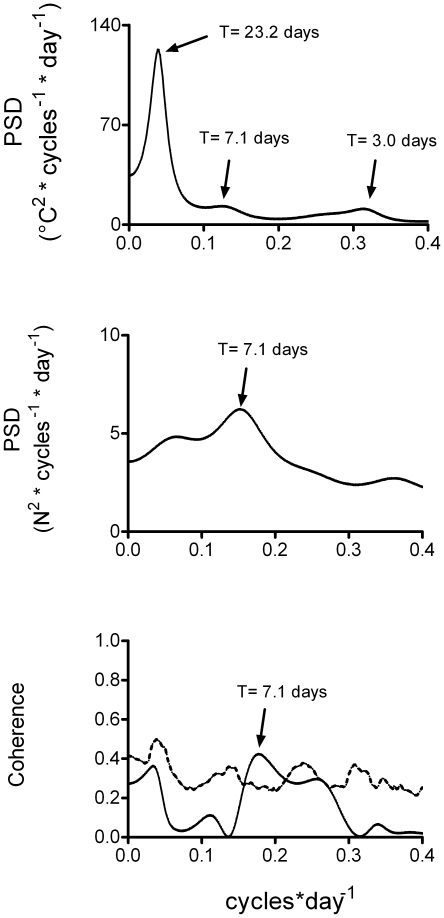
Frequency domain analysis of maximal air temperature variability (upper panel), of daily Emergency Department (ED) visits for syncope variability (middle panel) and of their relationship (coherence, lower panel). Broken line is the result of surrogate analysis. A major oscillatory component at 0.04 cycles×day^−1^ corresponding to a period of 23.2 days could be identified in the power spectrum of maximal air temperature variability. Two other minor oscillatory components were also present at 0.15 and 0.3 cycles×day^−1^, i.e. characterized by periods of ∼7 and ∼3 days, respectively. A significant non-random fluctuation in the pattern of ED visits for syncope (middle panel) was found at a peak frequency of 0.15 cycles×day^−1^ (period ∼7 days). As obtained from coherence and surrogate analyses, maximal temperature and syncope ED attendances variability were linearly coupled in a frequency range between 0.15 and 0.20 cycles×day^−1^ (between 7 and 5 days, respectively). This suggests a potential influence of maximal air temperature oscillations on the pattern of ED visits for syncope.

Figure 2 illustrates the power spectra of maximal temperature variability (upper panel), variability of ED visits for syncope (middle panel), coherence (lower panel) and the corresponding surrogate analyses (broken lines) from January 23^rd^ to July 31^st^. There were a significant oscillation (above the broken lines) at f_DO_ = 0.043 cycles^.^×day^−1^ (T_DO_ = 23.2 days) and two minor fluctuations at f_DO_ = 0.14 cycles^.^×day^−1^ (T_DO_ = 7.1 days) and f_DO_ = 0.31 cycles^.^×day^−1^ (T_DO_ = 3.0 days) in the power spectrum of maximal temperature variability. Power spectrum variability of ED visits for syncope showed a significant oscillation at f_DO_ = 0.14 cycles^.^×day^−1^ (T_DO_ = 7.1 days). These ∼7-day cycles did not appear to relate to the historical calendar week, in keeping with previous observations [20], [21].

Maximal temperature and syncope daily ED visits variability were significantly coupled in between 0.15 and 0.2 cycles×day^−1^ (i.e. from T_DO_ = 7 days to T_DO_ = 5 days). Phase analysis indicated that temperature changes preceded the changes in syncope occurrence by about a quarter of cycle, that is 1.5 days.

Power spectrum analysis of maximal temperature variability corresponding to the 3 epochs indicated that the large majority of temperature variability decline observed in June–July was due to a reduction of the power of the 23-day temperature oscillations (Table 3).

**Table 3:** Frequency domain analyses of Maximal Temperature spontaneous fluctuations (variability), during the three epochs.

	EPOCH 1	EPOCH 2	EPOCH 3
	Jan–Feb–Mar(69 days)	Apr–May(61 days)	Jun–Jul(61 days)
Max Temperature Variance °C^2^	20.8	13.0	6.9
Max Temperature DO_23_ °C^2^ (%)	14.9 (71.7)	8.4 (64.7)	3.5 (50.9)
Max Temperature DO_7_ °C^2^ (%)	2.0 (9.8)	1.1 (8.8)	1.8 (26.1)
Max Temperature DO_3_ °C^2^ (%)	1.5 (7.3)	2.6 (20.5)	0.4 (6.4)

Max Temperature Variance is the variance of the values of Maximal Temperature corresponding to each epoch. DO_23_ , DO_7_ and DO_3_ are the powers of Max Temperature rhythmic fluctuations with a period of ≈23, ≈7 and ≈3 days, respectively. (%) indicates % of total variance. Other abbreviations as in table 2.

## Discussion

In the present study we found no relationship between the progressive increase in temperatures from January to July and the number of ED visits for syncope. Unexpectedly, the months of June and July, characterized by the highest ambient air temperatures but the lowest maximal temperature variability, were associated with a lower rate of ED visits for syncope compared to the cooler months. We hypothesize that a reduced heat load, as it is the case when temperature variability is lower, may result in an enhancement of the orthostatic tolerance.

Finally, we found similar and related oscillatory patterns in both maximal temperature and syncope onset characterized by a periodicity of ≈7 days. These data suggest a potential influence of rhythmic changes in air temperature on the pattern of syncope occurrence.

### ED visits for syncope and temperature increase

The results of the present investigation suggest that ED visits for syncope were unaffected by the increase of maximal temperatures from January to July. This finding, while consistent with two previous studies [21], [22], challenged our first working hypothesis and seems to conflict with several pathophysiological studies indicating that heat exposure may facilitate syncope in humans particularly during standing [2]–[4], [23]. However, it must be pointed out that those investigations employed a more stressful stimulus, i.e. the sudden exposure of the human body to remarkable heat increases, compared to the mild day by day increase (and decrease) of maximal temperature as measured in the present investigation.

Our findings partially agree with the absence of effects of heat on cardiovascular hospital admissions reported in previous studies performed in London [24] , Madrid [25] and in 12 European cities from April to September [8] but do not overtly agree with the majority of other epidemiological observations [8], [10], [24], [25]. In those studies, however, a strict definition of syncope was missing and a heterogeneous group of heat-related diseases was considered.

In the individuals older than 75 years of our study (table 3), ED visits for syncope were unaffected by mild seasonal increases in the temperatures, in keeping with previous investigations indicating that the increase of morbidity [24], [25], i.e. hospital admission, was limited to the respiratory diseases [8].

In accordance with previous observations [1], [12] our results point to a higher female prevalence of ED visits for syncope compared to males (56% vs. 44%, respectively). Several gender differences in the cardiovascular control may account for this finding, including woman's smaller and less distensible left ventricle leading to a critical drop in stroke volume during orthostasis [26]. Since heat exposure may ultimately promote a decrease in central venous pressure [4], [5], one might expect women to be more sensitive to orthostatic syncope whenever temperatures are higher. Conversely, in our study, increases of temperature were not associated with different rate of women's ED visits for syncope (table 3), suggesting that a mild progressive increase of maximal air temperatures did not act as an additional critical stressor [27].

### Reduced frequency of ED visits for syncope during the hot months

In the present study the months of June and July showed the lowest values of maximal temperature variability and the lowest number of ED visits for syncope in relation to total ED visits, compared to the prior cooler months. Notably, these months had both the highest maximal temperatures and heat index values. Conversely, syncope onset was greater when there was greater variability in maximal temperature, i.e. in winter and spring, similarly to what is observed for the relationship between unusual temperature decreases, leading to high temperature variability, and the enhancement of myocardial infarction occurrence [28].

We do not have a definitive explanation for such an unexpected finding. It is possible that the lower the oscillations (variability) of maximal air temperature the lower the magnitude of daily heat loading that in turn might result in a decreased number/percentage of ED visits for syncope.

An additional possibility is that the larger temperature variability characterizing cooler months might promote a slow heat acclimatization phenomenon eventually contributing to the reduced number of syncope observed later, in June and July. In keeping with this hypothesis, a greater natural acclimatization to heat has been described in summer compared to winter in individuals who underwent a 20 minutes tilt-test during heat exposure at 40°C [29]. Accordingly, an improvement of orthostatic tolerance could be achieved in subjects after a 4-hour lasting heat exposure protocol that was repeated for 8 consecutive days [30] in order to accomplish a heat acclimatization. Conversely, the lack of a natural acclimatization, because of a sudden exposure to a large heat stress, might account for the reduced orthostatic tolerance observed in other human pathophysiological studies [2]–[4], [23].

In addition, observations derived from animal studies suggest that, after several days of heat exposure, there is a reprogramming of genes encoding for constitutive proteins and stress-inducible molecules [31] promoting an heat-acclimated phenotype. These genetic aspects of heat acclimatization are characterized by a memory [32]. It is conceivable that, whenever increases of temperature recur during the climate oscillations from winter to summer, the combination of the heat physiological modifications [4], [5] and the heat acclimatization memory [32] may result in a progressively more efficient human heat adaptation.

### Relationship between temperature and ED visits for syncope variability

In the present study the use of frequency domain analysis uncovered a relationship between variability of maximal air temperature and variability of ED visits for syncope. This relationship was hidden when temperature values and the number of ED visits for syncope were simply correlated, as also reported by previous studies [21], [22].

Indeed, variability of maximal temperature was characterized by rhythmic fluctuations with periods of ∼7 and ∼3 days and by a major oscillation with a period of ∼23 days. ED visits for syncope showed an oscillatory pattern with a period of ∼7 days. The assessment of a potential relationship between the two phenomena by coherence and surrogate analyses indicated a linear coupling between maximal temperature variability and the pattern of syncope ED attendances within a 5–7 day periodicity range and a peak at 7 days. Moreover, phase analysis indicated that temperature changes preceded the changes in syncope occurrence by about 1.5 days. Taken together these findings suggest a possible effect of ∼7-day rhythmic changes in temperature on the pattern of syncope onset.

Notably, a circaseptan (∼7 day) periodicity in blood pressure, heart rate [33] and tooth (striae of Retzius) [34] and bone growth rhythms [35] has been observed in both humans and animals [35]. This physiological long-term rhythm was hypothesized to be associated with the changes in the neural autonomic control regulating behavior and body development [34], [35]. In the present study, the finding of similar ∼7 day oscillations in air temperature raises the possibility of a potential influence of temperature fluctuations on human biological rhythms. However, ad hoc studies are needed to test this hypothesis.

### Limitations

Increases in ambient temperature do not occur in isolation. Indeed, not only they are associated with modifications in relative humidity, as we assessed by the heat index, but they also occur with changes in the barometric pressure, dew point and wind speed, often in a non-linear fashion. The potential role of these variables in affecting syncope occurrence was not taken into account in this study.

Humans may use multiple methods to adapt to high environmental temperatures [20] including staying indoors, wearing cooler clothing, increasing fluids intake or by using air conditioning. In the present study we could not assess changes in these variables, and their contribution to the lower rate of ED admissions for syncope during the two hot months, i.e. June and July.

Changes in the alimentary habits consistent with an increase of water ingestion may take place during the hot season. Water intake acutely improves orthostatic tolerance in healthy subjects [36], [37], presumably by osmosensitive mechanisms [38] which reflexly increase blood pressure. This might eventually result in a reduced number of syncope visits to the ED during summer.

Air conditioning was found to reduce mortality in urban areas [39] during heat waves [20] but its effects on general morbidity and ED admissions are unknown during mild increases of temperature. Air conditioning may also theoretically interfere with human physical adaptation to heat and actually promote syncope unless the subjects were constantly living in an air conditioned environment. This is unlikely with the present population as the use of air conditioning within inhabitants of northern Italy is highly irregular.

Finally, the design of the present study was aimed at detecting a potential effect of temperature increases on syncope by focusing on the number of ED visits due to syncope. It is possible that people may consider syncope to be a frequent, non life-threatening event particularly in summer. Thus, we cannot exclude the possibility that some patient with syncope either did not see a doctor or were seen only in out-patient clinics instead of going to ED [40] and that their health care pattern was different in the cooler months.

### Conclusions

The results of the present study challenge the ordinary belief that syncope is promoted by high environmental temperatures. Indeed, the rate of ED visits for syncope was lower during the hottest months compared to spring and winter periods.

Also, syncope onset was independent of the absolute values of maximal air temperature. Instead, as observed in our temperate zone of the northern hemisphere from January to May, a higher maximal air temperature variability was found to be associated with an increased rate of ED admissions for syncope. This observation may be critically important in terms of public health policy. If syncope is more frequent in the winter and spring months, there may be a social imperative to provide warnings and increase the availability of health related facilities at these times in order to reduce the clinical and social consequences of the loss of consciousness and falls.

Finally, maximal temperature ≈7-day rhythmic fluctuations were found to be correlated with similar oscillations in the pattern of syncope ED admissions. Therefore, air temperature seems to affect the pattern of syncope onset by the 7-day non-random oscillatory component that is part of the maximal temperature spontaneous variability.
